# Time-Domain Simulation and Optimization of the Memory Window for HZO-Based FeFETs Using the NLS Model

**DOI:** 10.3390/mi17070828

**Published:** 2026-07-10

**Authors:** Shangda Han, Weifeng Lü, Yekun Liang, Tianyu Dai

**Affiliations:** 1 School of Electronic Information, Hangzhou Dianzi University, Hangzhou 310018, China; 2 Zhejiang Key Laboratory of Intelligent Vehicle Electronics Research, Hangzhou 310018, China; 3 School of Electronic Information, Wuhan University, Wuhan 430072, China; yekun_liang@whu.edu.cn

**Keywords:** ferroelectric transistors, nucleation-limited switching (NLS), fully depleted silicon-on-insulator (FDSOI), Monte Carlo simulation, memory window

## Abstract

Hafnium-zirconium oxide (HZO)-based ferroelectric field-effect transistors (FeFETs) are expected to become core devices for new embedded memory and compute-in-memory systems. However, existing simulations rely on finite-element-based TCAD tools, which are computationally intensive and time-consuming, and they struggle to account for the dynamic flipping of ferroelectric domains. This paper utilizes a time-domain simulation framework based on the nucleation-limited switching (NLS) model coupled with the surface potential of a MOSFET, enabling a self-consistent solution for polarization and electrical characteristics; a Monte Carlo method is employed to simulate device variability, and Shmoo plots are used to identify optimal programming and erasure process windows; an integrated solution is proposed for 22 nm FDSOI devices, addressing geometric scaling, modification of the Landau–Khalatnikov (L-K) dynamic model for ultrathin ferroelectric layers, and suppression of short-channel effects. Model validation is limited to selected operating metrics, and predictive accuracy outside the calibrated cases requires additional independent datasets. This method enables end-to-end simulation of FeFETs, from material polarization and device electrical characteristics to performance optimization, thereby providing model-based analytical and design support for the development of advanced, ultra-low-power FeFETs.

## 1. Introduction

The physical separation of memory and computing units in the von Neumann architecture has led to severe “memory wall” and “power wall” issues in integrated circuits, which have become a core bottleneck limiting the development of high-performance computing and edge AI chips. Compute-in-memory, which performs logical operations within the memory unit to eliminate the power consumption and latency caused by data movement, is considered the key technological path to overcoming this bottleneck. Among various novel devices, hafnium-based ferroelectric field-effect transistors (FeFETs) rely on spontaneous polarization as their physical foundation and offer advantages such as non-volatile storage, high-speed read/write capabilities, low operating voltage, and high integration density. Furthermore, their core dielectric—hafnium zirconate (HZO) thin films is fully compatible with standard CMOS processes, enabling large-scale integration [1,2,3,4], and is expected to become core devices for embedded memory, neuromorphic synaptic devices, and compute-in-memory chips [5,6,7,8,9,10,11,12]. Although the present framework is developed for HZO-based FeFETs, its separation of ferroelectric switching and semiconductor current calculation could be extended to non-HZO systems, including ScAlN/AlGaN/GaN ferroelectric HEMTs, provided that the polarization parameters, switching-field distribution, dielectric response, and channel-transport model are recalibrated for the target material system [13].

The electrical performance and reliability of FeFETs are highly dependent on the synergistic optimization of the polarization reversal kinetics of the ferroelectric layer, the charge-coupling characteristics at the ferroelectric–semiconductor interface, and device structural parameters. Currently, simulation analyses of FeFETs suffer from three significant shortcomings: (1) Simulation models primarily focus on fitting static electrical characteristics, lacking time-domain dynamic evolution analysis of ferroelectric domains under pulsed excitation, and thus cannot accurately describe the polarization reversal rates and the establishment of the memory window (MW) during programming/erasing processes. (2) They neglect device variability caused by grain size in polycrystalline HZO films and fluctuations in the activation field distribution [4,14], resulting in simulation results that are only applicable to ideal single-device models and cannot guide yield optimization for mass production. (3) Existing models rely on complex and time-consuming finite-element TCAD simulations; while ensuring accuracy, they are unsuitable for large-scale circuit analysis. Furthermore, charge-matching theory indicates that a mismatch between the polarization charge in the ferroelectric layer and the semiconductor channel charge can induce high electric fields and charge traps at the interface [4,12,15], which is the core cause of device endurance degradation and retention failure [1,11,15]. Precise time-domain coupled simulations are a necessary prerequisite for quantifying charge matching and optimizing interfacial characteristics [6,7,14,16,17]. In this compact workflow, interface traps, wake-up, fatigue, retention loss, and imprint are treated as reliability constraints rather than as time-evolving internal state variables.

To address the aforementioned challenges, this paper systematically conducts a time-domain simulation analysis of nanometer HZO-based FeFETs. First, a full-process time-domain simulation framework for FeFETs, based on the NLS model [11], is adopted to enable the simultaneous time-domain solution of polarization dynamics and device electrical characteristics. Second, the Monte Carlo method was introduced to quantify process variability, and a Shmoo-chart-based process window standardization extraction workflow was used [10], with a focus on model refinement and parameter optimization for a 22 nm FDSOI transistor to address simulation inaccuracies caused by ultrathin ferroelectric layers and short-channel effects. Finally, we analyzed the regulatory mechanisms of pulse parameters, material parameters, and structural parameters on the MW, switching current ratio, and subthreshold characteristics, and adopted a multidimensional performance optimization scheme. Compared with the previously reported NLS-FeFET implementation, this work focuses on literature-calibrated time-domain simulation, device-level variability analysis, and process-window extraction for scaled HZO FeFET operation. The simulation results provide preliminary model-based guidance for structural design, process tuning, and advanced-node analysis of nanoscale FeFET devices.

## 2. Device Structure and Simulation Models

### 2.1. FeFET Device Structure and Core Operating Principles

**Figure 1:**
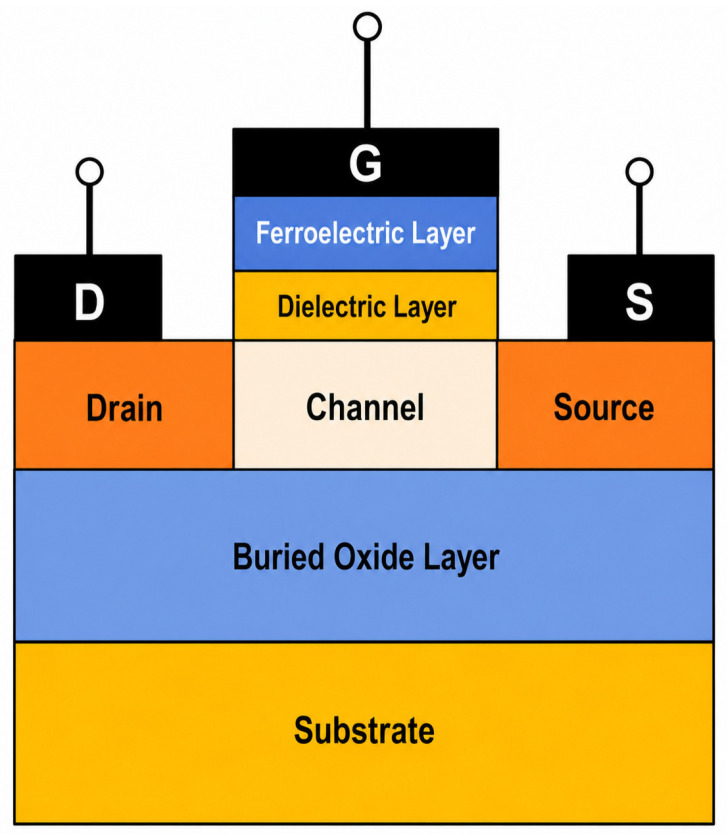
Schematic diagram of the MFIS stacked structure for FDSOI FeFET devices. The stack shows the ferroelectric/insulator/semiconductor charge-coupling path used in the compact model; no internal floating metal electrode is included.

Figure 1 shows a cross-sectional view of an FeFET device employing a metal–ferroelectric–insulator–semiconductor (MFIS) stack structure [1,6,11,18,19]. The compact simulations do not include an internal floating metal electrode, so no separate metal thickness, work function, or floating-electrode charge equation. Its core operating mechanism is the threshold voltage modulation effect of ferroelectric polarization [9]: when a forward programming voltage is applied to the gate, the polarization direction of the ferroelectric layer points toward the channel, inducing carrier accumulation in the channel and causing the device threshold voltage to shift positively; when a reverse erasure voltage is applied, the polarization direction reverses, causing the threshold voltage to shift negatively. The difference in threshold voltage between these two polarization states is known as the memory window (MW) [4,8,20], which directly determines the read margin and noise immunity of the device [14].

The MW can be estimated from the coercive field, thickness, dielectric constant, and polarization of the ferroelectric layer as follows [4,6,7,12,21]:

(1) $$MW\approx 2E_{c}\cdot T_{FE}\left[1-\frac{2E_{c}\cdot \varepsilon_{FE}\cdot \varepsilon_{0}}{P_{s}\cdot \ln\left(\frac{1+P_{r}/P_{s}}{1-P_{r}/P_{s}}\right)}\right]$$

In this equation, *E*_c_ represents the macroscopic coercive field of the HZO ferroelectric layer; *T*_FE_ represents the physical thickness of the ferroelectric layer; *ε*_FE_ represents the relative permittivity of the ferroelectric layer; *ε*_0_ represents the permittivity of free space; *P*_s_ represents the spontaneous polarization; and *P*_r_ represents the remanent polarization. This formula clarifies the quantitative influence of material and structural parameters on the memory window, providing a theoretical basis for optimizing device performance. Equation (1) provides a first-order MW estimate under a quasi-static charge-matching assumption; dynamic trap generation, wake-up, fatigue, retention degradation, and imprint are outside the validity range of this closed-form expression.

### 2.2. NLS Model Framework for FeFET Simulation

The simulation platform employs a hierarchical modular design consisting of three core modules: the ferroelectric domain-dynamics layer, the semiconductor electrical-calculation layer, and the charge-coupled iteration layer. This structure enables automated time-domain simulation. The ferroelectric layer is described by the NLS model, which captures the stochastic switching of multiple domains, as expressed in Equations (2) and (3) [22]:

(2) $$p(t,E)=1-\exp\left(-t\cdot f_{0}\cdot \exp\left(-\frac{E_{a}-E}{E_{0}}\right)\right)$$

where *p*(*t*,*E*) is the probability that a single ferroelectric domain switches under an electric field *E* at time t, *f*_0_ is the trial frequency, *E*_a_ is the activation field for domain switching, and *E*_0_ is an empirical electric-field acceleration parameter with units of electric field rather than the thermal voltage *k*_B_*T*/*q*.

(3) $$P(t)=P_{r}\cdot \int_{0}^{\infty }\left[1-2p(t,E)\right]\cdot f(E_{a})\;dE_{a}$$

Here, *f*(*E*_a_) is the probability density function of the activation field. The mean and standard deviation of this activation-field distribution are used to represent the average switching field and HZO grain-level field dispersion, respectively, while *P*_r_ and the characteristic switching time are linked to measurable *P*-*E* and pulse-switching responses.

The semiconductor layer uses a surface-potential-based MOSFET model to calculate the drain current over the full operating range, including the subthreshold, linear, and saturation regions, as shown in Equation (4) [22]:

(4) $$I_{D}=\mu \frac{W}{L}C_{ox}\left[\left(\psi_{s}-\psi_{d}\right)+V_{T}\left(e^{-\frac{\psi_{s}}{V_{T}}}-e^{-\frac{\psi_{d}}{V_{T}}}\right)\right]$$

Here, *μ* is the carrier mobility; *W*/*L* is the channel width-to-length ratio; *C*_ox_ is the gate-oxide capacitance per unit area; *ψ*_s_ and *ψ*_d_ are the source and drain surface potentials, respectively, and the difference between these two quantities represents the potential difference across the channel; *V*_T_ is the thermal voltage. For the 22 nm FDSOI case, Equation (4) is used as a compact current expression calibrated over the target bias range, rather than as a full quantum-corrected short-channel TCAD transport model.

The two-layer model performs self-consistent iteration through the charge-conservation equation shown in Equation (5) [7,17,19,21], which ensures real-time matching between the voltage partitioning and polarization of the ferroelectric layer and the channel surface potential and carrier concentration.

(5) $$D(t)=\varepsilon_{0}\varepsilon_{FE}E_{FE}(t)+P(t)=Q_{s}(t)+Q_{trap}(t)$$

During transient compact simulation, the polarization term is updated from the NLS time-domain state, whereas the semiconductor charge and trap-charge terms are evaluated at each time step using a quasi-static charge-balance approximation.

To quantify the device dispersion caused by process variations, this paper introduces a Monte Carlo random sampling method: 1000 activation-field samples are drawn from a normal distribution with mean a and standard deviation *b*, and 100 independent device samples are simulated in parallel. The resulting samples are used to statistically analyze the distribution patterns of the storage window, threshold voltage, and on/off ratio [4,6,17], thereby addressing the industry-wide challenge of multi-device consistency analysis. The samples are treated as independent device-level realizations; spatial correlation and cycle-to-cycle trap evolution are not included in the present compact workflow. The simulation environment temperature is set to 300 K, with a p-type silicon-doped channel. The core simulation parameters are shown in Table 1; these values define the manuscript-level simulation setup.

### 2.3. 22 nm FDSOI Process Adaptation Plan

For 22 nm gate-length fully depleted silicon-on-insulator (FDSOI) FeFETs, we propose a three-step adaptation scheme comprising “geometric scaling, material modification, and electrical optimization” to address the issues of polarization distortion in ultrathin ferroelectric layers and a surge in short-channel leakage current under advanced process conditions [4,6,8,10,12]. Geometric scaling: Using a mature 28 nm process as the baseline, the gate length and other geometric parameters such as ferroelectric layer thickness and channel thickness are uniformly scaled down by a factor of 0.7857; this factor is used as a compact-model geometry adaptation for the present simulation target and should be interpreted as a first-order design approximation rather than a process-qualified scaling rule. Ultra-thin ferroelectric layer material correction: For a 1.8 nm HZO film, the Landau–Khalatnikov (L-K) kinetic model is parameterized using the listed L-K coefficients to describe the nonlinear *P*-*E* response of the ultrathin ferroelectric layer. Suppression of short-channel effects: The channel doping concentration was reduced, and the flat-band voltage and drain bias were optimized to suppress the drain-induced barrier lowering (DIBL) effect and subthreshold leakage. The geometric parameters are summarized in Table 2, and the corresponding HZO L-K, semiconductor, and bias parameters are listed in Table 3. Using this scaling procedure as a predictive process-design method would require further electrostatic validation with calibrated TCAD or measured 22 nm devices.

## 3. Simulation Results and Discussion

### 3.1. Calibration of the Memory Window Model and Quantitative Error Analysis

**Figure 2:**
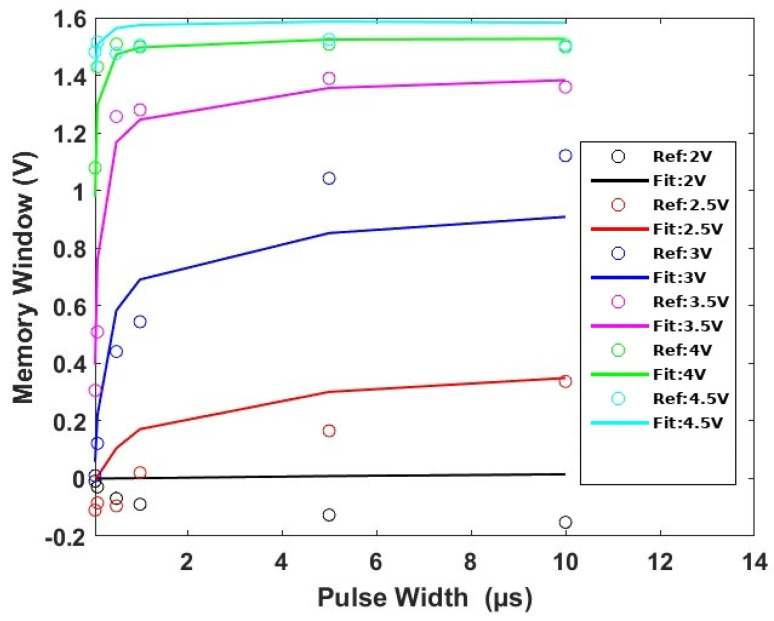
Calibration of the compact NLS-FeFET workflow using reference MW points under different pulse amplitudes and pulse widths [22]. The symbols denote the reference MW points used in the Scalable-FeFET workflow, and the curves show the fitted compact-model response.

Based on the HZO material-level parameter calibration, the storage-window fitting was benchmarked against the reference MW points used in the Scalable-FeFET compact-model workflow associated with Deng et al. [22]. This comparison provides a calibration reference for the compact NLS-FeFET workflow and verifies that the fitted response follows the reported MW trend. The simulated curves reproduce the monotonic increase in the storage window with pulse amplitude and pulse width, consistent with electric-field-time coordinated switching in the NLS model. The corresponding calibration results are shown in Figure 2.

The quantitative error analysis shows larger relative errors in the low-voltage regime, where the reported MW approaches zero, and the relative-error denominator becomes small. Within the reported FeFET operating range, representative errors are 0.43% at a 3.5 V pulse, 1.19% at a 4.0 V pulse, and 4.07% at a 4.5 V pulse. These values indicate calibration-level agreement at selected operating points, but they do not establish a global accuracy bound over all bias and reliability conditions.

### 3.2. Monte Carlo Convergence and Parameter Sensitivity

**Figure 3:**
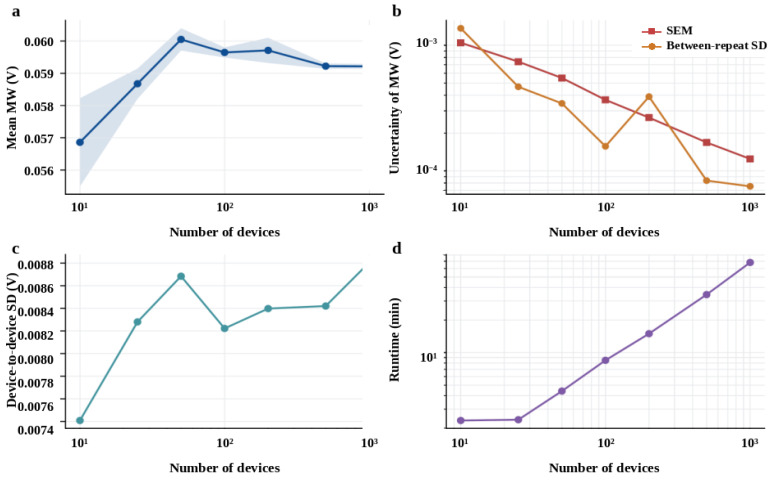
Monte Carlo convergence of the simulated MW statistics. (**a**) Mean MW as a function of the number of simulated devices; (**b**) statistical uncertainty of MW, including SEM and between-repeat SD; (**c**) device-to-device standard deviation; (**d**) runtime as a function of the number of simulated devices.

Monte Carlo convergence was evaluated using 10, 25, 50, 100, 200, 500, and 1000 device samples, with five repeated runs at each sample size. As shown in Figure 3, the mean MW changes from 0.0569 V at 10 devices to 0.0592 V at 1000 devices, while the standard error of the mean decreases from 1.05 × 10^−3^ V to 1.25 × 10^−4^ V. The corresponding 88.1% reduction in statistical uncertainty indicates that the Monte Carlo workflow provides stable variability estimates for the present compact simulation.

**Figure 4:**
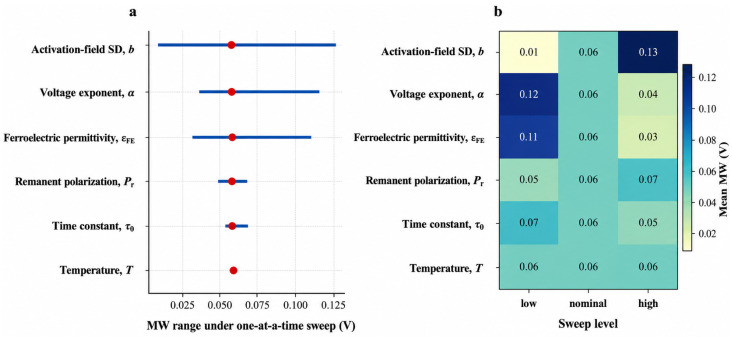
One-at-a-time sensitivity analysis of the simulated MW around the nominal parameter set. (**a**) MW range under one-at-a-time parameter sweeps; (**b**) heatmap of MW values at low, nominal, and high sweep levels. The activation-field distribution width and field-acceleration exponent produce the largest MW changes, indicating the need to specify the HZO switching-field distribution carefully.

A one-at-a-time sensitivity analysis around the nominal parameter set was used to identify the dominant controls on the simulated MW. Figure 4 shows that the activation-field distribution width *b* gives the largest MW variation range of 0.1178 V, followed by the voltage acceleration exponent *α* with a range of 0.0795 V and the ferroelectric relative permittivity *ε*_FE_ with a range of 0.0789 V. By contrast, sweeping the temperature from 250 K to 350 K changes the MW by only 0.00123 V under the present parameter set. These results identify switching-field dispersion and field-acceleration kinetics as the dominant parameters in the present NLS-based compact workflow.

### 3.3. Verification of Device Transfer Characteristics and Switching Performance

**Figure 5:**
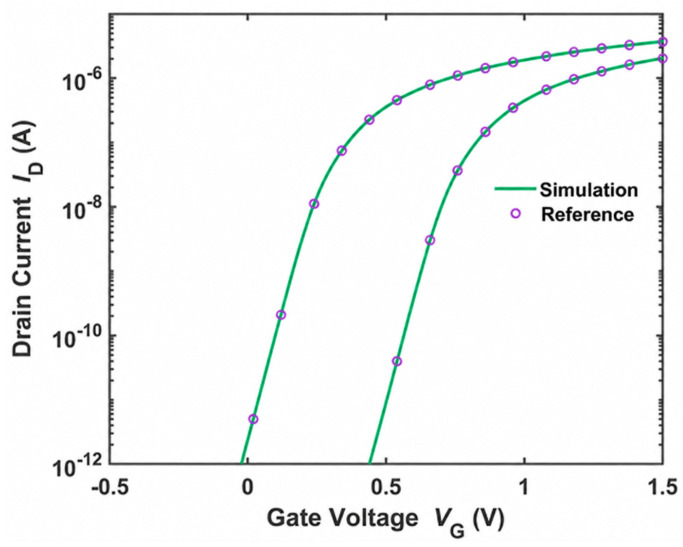
Simulated programming/erasing state characteristics of HZO-based FeFETs, obtained with the compact-model workflow calibrated from Ref. [22]. The separated programmed and erased branches correspond to polarization-induced threshold-voltage modulation.

**Figure 6:**
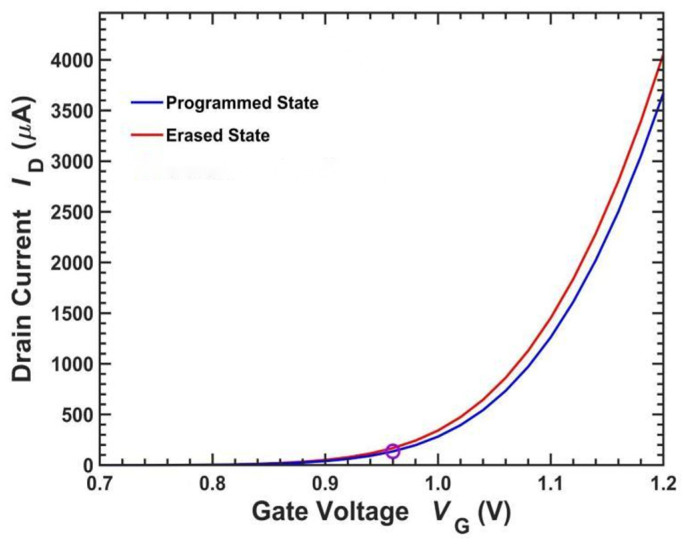
*I*_D_-*V*_G_ curves of FeFETs under programming/erasing conditions. The curves show read-current separation between the two polarization states under the compact-model readout condition; the on/off ratio is extracted from the corresponding read-current arrays rather than from visual inspection of Figure 5.

Based on the calibrated parameters, the transfer characteristics (*I*_D_-*V*_G_) of the programmed and erased FeFET states were simulated, as shown in Figure 5. Figure 6 presents the corresponding read-current curves after programming and erasing. Together, the two figures describe complementary outputs of the compact model: Figure 5 shows polarization-induced threshold-voltage modulation, whereas Figure 6 gives the read-bias current separation used for the on/off ratio extraction. The on/off ratio is calculated from the saved read-current arrays under the selected read-bias rule. The simulated curves follow the reference characteristics across the plotted bias range, including the subthreshold, linear, and saturation regions, and reproduce the hysteresis trend associated with ferroelectric polarization modulation.

At the selected read bias, the simulated transfer curves show clear separation between the programmed and erased states. Because the extracted on/off ratio is sensitive to the numerical floor of the simulated off-state current, the readout result is reported as a compact-model read-margin metric, not as an experimentally demonstrated ultra-high current ratio. For process-window screening, the on/off ratio is extracted from saved read-current arrays generated by the compact MATLAB R2024a workflow. Cases with extremely small simulated *I*_off_ are treated as raw numerical ratios and are used only to test whether the *I*_on_/*I*_off_ > 10^6^ read-margin criterion is satisfied. This result corresponds to a simulated read margin under the selected bias condition; endurance, retention, and stress-cycle reliability require additional validation beyond this static readout comparison.

### 3.4. FDSOI FeFET Performance Validation

**Figure 7:**
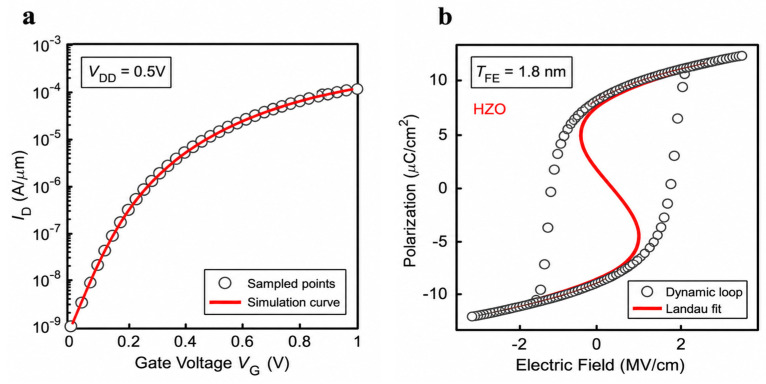
Simulated 22 nm HZO-FeFET characteristics obtained with the FDSOI/L-K parameter set derived from Refs. [23,24,25]. (**a**) Transfer characteristic curve; (**b**) *P*-*E* hysteresis loop of the HZO ferroelectric layer. The simulation evaluates whether the compact workflow reproduces the expected transfer and polarization trends for the scaled device.

To verify the model’s advanced-node adaptation capability, the transfer characteristics of a 22 nm gate-length FDSOI FeFET and the *P*-*E* loop of an ultrathin HZO layer were simulated with an FDSOI/L-K parameter set derived from the literature. The results are shown in Figure 7. Ref. [22] supports the MW/NLS compact-model calibration discussed in Section 3.1, whereas Refs. [23,24,25] provide the device and material basis for the FDSOI/L-K simulation in Figure 7, including the negative-capacitance FET body-biasing configuration, laminated HSO/HZO FeFET memory behavior, and doped-HfO_2_ ferroelectric response. For the transfer-characteristic comparison, the relative error in drain current remains below 5% across the gate-voltage range of 0~1 V. The simulated subthreshold swing is 68 mV/dec, corresponding to a 4.6% difference from the reported reference value of 65 mV/dec. The simulated and reference *I*_off_ values differ by 3.8% within the selected literature-parameter operating window.

In terms of polarization characteristics, the *P*-*E* curves of the 1.8 nm ultrathin HZO layer follow the reported reference trend, with the source figure reporting a maximum error of 2.5% in polarization intensity and errors of 2.8% and 2.7% in the positive and negative coercive fields, respectively. The L-K coefficients in Table 3 are used here as adopted model parameters for reproducing the reported *P*-*E* response. The 1.8 nm HZO case is therefore used as a compact-model/literature-parameter case for FDSOI scaling analysis, rather than as evidence for newly measured ferroelectricity in an independently fabricated 1.8 nm HZO film. These coefficients are not independently extracted from raw *P*-*E* numerical data in the present manuscript. The L-K damping parameters successfully reproduced the smooth transition characteristics near the coercive field, eliminating the issue of numerical discontinuities in simulations of ultrathin layers. The sub-5% error statement is limited to the reported transfer-characteristic and *P*-*E* metrics in this literature-based FDSOI comparison, rather than to all MW, variability, or reliability conditions.

Computational cost was characterized by recording representative runtimes of the Python 3.12 implementation and comparing them with representative reports from the TCAD-acceleration literature. As summarized in Table 4, MW validation and Monte Carlo convergence required 0.526 h and 2.254 h, respectively, while a representative expanded Shmoo point required 0.036 h. These values provide runtime context for compact-model parameter screening. They are not a direct replacement for calibrated commercial TCAD, which resolves more detailed electrostatic and transport physics.

### 3.5. Shmoo Analysis of Process Windows and Extraction of Optimal Parameters

A full-parameter sweep was performed to analyze the programming/erasing behavior of the device MW. Pulse amplitudes ranged from 2.0 to 3.0 V, and pulse widths ranged from 1 to 11 μs, giving 36 parameter combinations. The key simulation parameters are listed in Table 5. Using a switching ratio of *I*_on_/*I*_off_ > 10^6^ and an on-state current of *I*_on_ > 100 μA as the pass criteria, 13 qualified parameter combinations were identified, corresponding to a process-window yield of 36.1%. Table 6 summarizes the dependence of the switching ratio on pulse parameters. At low amplitudes, polarization reversal is insufficient, and no qualified combinations are obtained. When the amplitude is at least 2.6 V, the switching ratio increases sharply. At a fixed amplitude, a longer pulse width leads to more complete polarization reversal and a higher switching ratio.

**Figure 8:**
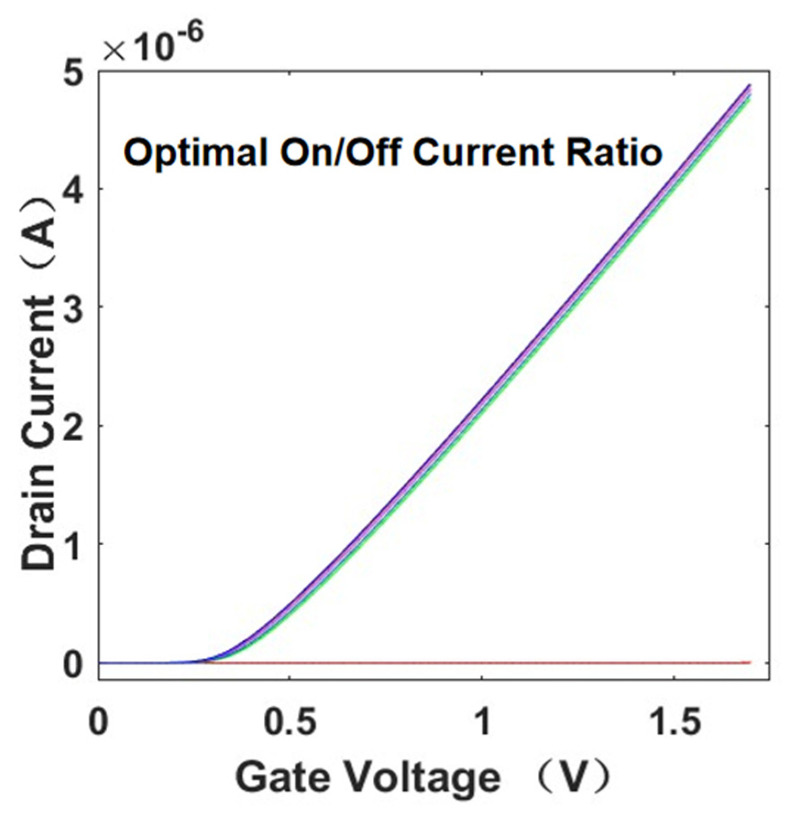
Device transfer characteristics at the optimal on/off current ratio. All colored lines correspond to transfer curves of five individual devices. The selected pulse condition produces a clear read-current separation between the programmed and erased states.

**Figure 9:**
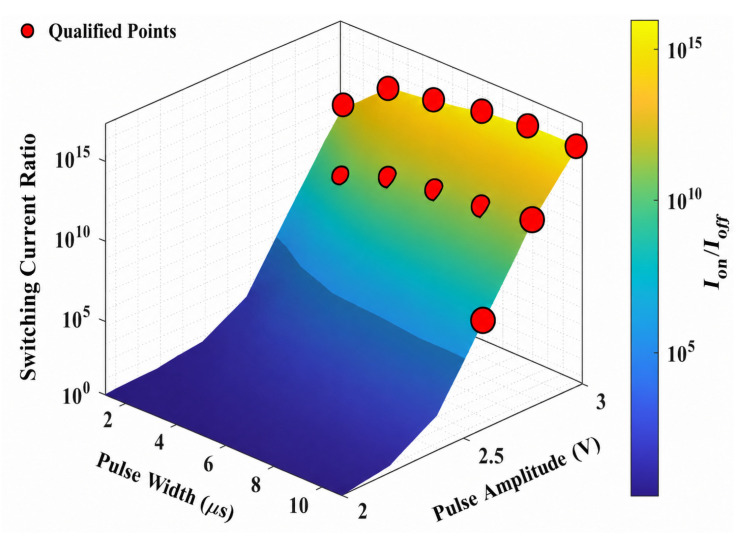
FeFET device Shmoo qualification analysis surface. The qualified region is concentrated at higher pulse amplitudes and longer pulse widths, where polarization reversal is more complete.

The optimal process parameters are a pulse amplitude of 3.0 V and a pulse width of 11 μs. Under these conditions, the compact simulation yields the largest read-margin metric within the swept pulse window, satisfying the prescribed *I*_on_/*I*_off_ > 10^6^ and *I*_on_ > 100 µA screening criteria. In this region, very large raw ratios arise when the simulated off-state current becomes extremely small. They are therefore interpreted as compact-model screening metrics, not as experimentally demonstrated transistor on/off ratios. Figure 8 shows the transfer characteristic curves of the devices at the optimal on/off current ratio. This figure displays the transfer characteristic curves of five devices at the optimal on/off ratio, with the red curve representing the transfer curve in the off state. The Shmoo heatmap is shown in Figure 9, where the qualified region is concentrated in the high-amplitude, long-pulse-width range [4]. This visually illustrates the feasible range of process parameters and supports simulation-level pulse-parameter tuning.

**Table 6:** Table showing the effects of pulse amplitude and pulse width on the switching ratio. The table summarizes the qualitative trend used to identify the pulse window in Figure 9.

Conditions	Results
Pulse amplitude < 2.4 V	The switching ratio is low, and most combinations fail to meet the pass criteria.
Pulse amplitude ≥ 2.6 V	The compact-model on/off ratio increases sharply; some raw simulated ratios exceed the 10^6^ criterion, especially when the simulated off-state current approaches the numerical floor.
The wider the pulse width	At the same amplitude, a wider pulse produces more complete polarization reversal and a higher on/off ratio.
High amplitude and long pulse width	The strongest read-margin condition appears at amp = 3 V and pw = 11 µs; the raw compact-model ratio is used as a screening metric for the 10^6^ pass criterion.

### 3.6. Multi-Parameter Control Mechanisms and Engineering Optimization Strategies

**Figure 10:**
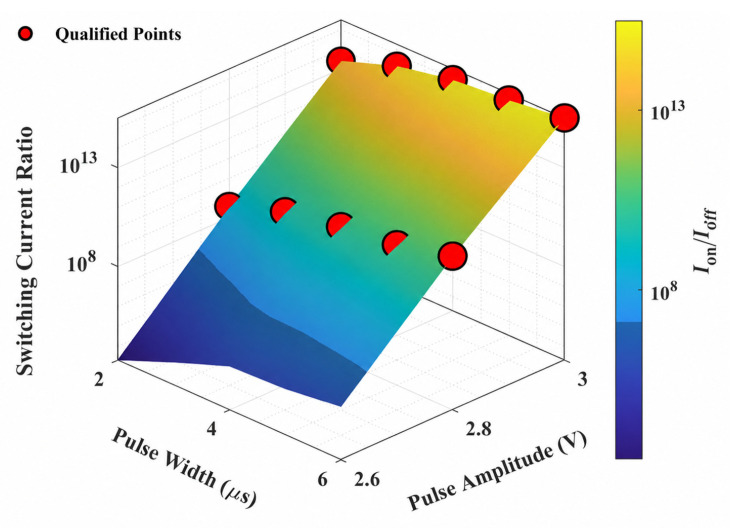
Three-dimensional Shmoo qualification analysis for pulse-optimized FeFETs. The surface representation shows how the pass/fail boundary depends on pulse amplitude and pulse width.

Based on the results of the comprehensive simulation, an integrated optimization strategy for FeFET performance can be proposed across three dimensions: pulse, material, and structure. At the pulse parameter level, an amplitude of 2.6~3.0 V and a pulse width of 2~6 μs are selected to balance switching efficiency and power consumption [2]. The same pulse-window trend is also shown as a three-dimensional surface in Figure 10, providing a complementary view of the pass/fail boundary without changing the underlying Shmoo criteria.

A quasi-static trap-density loading analysis links the charge-matching discussion to an interface-trap scale through *Q*_trap_ = *qN*_it_ and ∆*V*_FB_ = *qN*_it_/*C*_ox_. As shown in Table 7, increasing *N*_it_ from 1 × 10^12^ cm^−2^ to 5 × 10^12^ cm^−2^ raises the equivalent flat-band shift from 0.017 V to 0.084 V, while the corresponding trap-charge loading remains within 3.20% of *P*_r_. This analysis quantifies the charge scale associated with interface-quality control without introducing a new dynamic trap-evolution model.

At the material-parameter level, grain-size optimization can narrow the activation-field distribution, improve domain-switching consistency, and regulate remanent polarization to balance the MW and charge matching [8,10,17]. At the structural-parameter level, ultrathin ferroelectric layers of 1~10 nm can enhance the internal electric field [7,26], whereas reduced channel thickness and optimized gate length improve electric-field concentration. Lower channel doping can suppress short-channel effects and drain-barrier lowering, and optimization of the insulating-layer thickness and interfacial quality can reduce charge trapping [1,9,15,27]. This strategy is expected to improve the simulated MW and on/off ratio, whereas quantitative prediction of endurance and retention requires explicit trap-evolution and stress-cycle modeling.

## 4. Conclusions

This paper addresses the core challenges of FeFET devices based on HZO—namely, the lack of time-domain simulation, the difficulty of quantifying process variability, and poor adaptation to nanoscale nodes. By applying the NLS-FeFET coupled time-domain simulation platform, the authors successfully performed device electrical characteristic simulations, storage window extraction, and process adaptation optimization for the 22 nm FDSOI process. The coupled simulation model reproduces the reported MW and transfer-characteristic trends within the calibrated operating cases; the Monte Carlo method enables quantitative analysis of device variability, and the Shmoo plot identifies a candidate programming/erasing condition of 3.0 V/11 µs for process-window assessment; the integrated adaptation scheme offers an exploratory compact-model route for analyzing ultra-thin ferroelectric layers and short-channel effects in 22 nm FDSOI. The convergence analysis shows that increasing the Monte Carlo sample size from 10 to 1000 devices reduces the MW standard error by 88.1%, and the sensitivity analysis identifies the activation-field distribution width as the dominant variability parameter. The runtime context further indicates that the compact workflow is suitable for parameter screening and variability studies before more detailed TCAD verification. The sub-5% error statement is restricted to the reported FDSOI transfer-characteristic and *P*-*E* comparison metrics, and the model requires further independent validation before it can be used as a fully predictive advanced-process design tool.

The present compact workflow does not explicitly resolve dynamic trap generation, trapping/detrapping kinetics, wake-up, fatigue, retention degradation, or imprint. Future research will expand the physical models to account for ferroelectric layer defect evolution and dynamic charge trap changes, incorporating reliability characteristics such as fatigue, wake-up, and retention failure to enable full device lifecycle simulation. Concurrently, we will conduct 3D device structure simulations, integrate with TCAD tools, and perform circuit-level co-simulation to support the future development of HZO-based FeFETs in advanced compute-in-memory chips.

## Figures and Tables

**Table 1:** Device core parameters table. The table lists the nominal NLS and transfer-function parameters used in the manuscript-level simulation setup.

Key Parameters of the Ferroelectric Layer		Transfer Function Simulation Parameters	
Parameter Name	Value	Parameter Name	Value
Mean of the activation field distribution *a*	1.60 MV/cm	Flat-band voltage *V*_FB_	−0.5 V
Standard deviation of the activation field distribution *b*	0.35 MV/cm	Programming/erasing pulse amplitude amp	1.9 V
Residual polarization intensity *P*_r_	17 μC/cm^2^	Pulse width *pw*	2 μs
Domain-switching characteristic time constant *τ*_0_	1.9 × 10^−8^ s	Pulse interval *delay*	10 μs
Electric field acceleration factor *α*	3.0	Simulation time step *t*_step_	2 × 10^−8^ s
Probability shape parameter *β*	2.0	Pulse rise/fall time	1 × 10^−9^ s
Relative permittivity of the ferroelectric layer *ε*_FE_	25.0		

**Table 2:** Geometric parameters of FDSOI FeFET devices. These parameters define the compact FDSOI geometry used for the 22 nm adaptation analysis.

Parameter Name	Value
Geometry scaling factor scalegeo	0.7857
Gate length *L*_g_	22 nm
HZO ferroelectric layer thickness *T*_FE_	1.8 nm
Gate oxide thickness *T*_ox_	0.36 nm
Channel silicon layer thickness *T*_si_	2.18 nm
Buried oxide layer thickness *T*_box_	9.09 nm
Substrate thickness *T*_sub_	36.36 nm

**Table 3:** HZO ferroelectric layer kinetic parameters and FeFET semiconductor and bias parameters. The listed L-K and semiconductor parameters are used for the FDSOI transfer-characteristic and *P*-*E* comparison.

Parameter Name	Value	Parameter Name	Value
First-order Landau potential coefficients *α*_HZO_	−2.17 × 10^11^ cm/F	Source-drain junction doping concentration *N*_D_	10^20^ cm^−3^
Second-order Landau potential coefficients *β*_HZO_	3.01 × 10^21^ cm^5^/F/C^2^	Doping concentration at the channel bottom *N*_A_	10^15^ cm^−3^
L-K kinetic damping *ρ*_LK_	3.0 × 10^5^	Drain bias voltage *V*_DD_	0.5 V
Relative permittivity of the ferroelectric layer *ε*_FE_	25	Flat-band voltage *V*_FB_	−0.50 V
Effective coupling coefficient *η*_eff_	0.15	Low-field carrier mobility *μ*	50 cm^2^/(V·s)

**Table 4:** Runtime context for the compact simulation workflow, with a comparison to TCAD-related literature.

Case	Runtime or Comparison Metric	Interpretation
MW validation	0.526 h	Compact-model validation
Monte Carlo convergence	2.254 h	Variability statistics
Expanded Shmoo, representative point	0.036 h	Single compact sweep point
Expanded Shmoo, cumulative points	11.921 h	Large parameter screening
ML-TCAD literature context	13,600× acceleration reported for FeFET reliability analysis [26]	Literature context; not a local TCAD run

**Table 5:** Key Parameters of Pulse and FeFET Devices. The Shmoo pass criteria are *I*_on_/*I*_off_ > 10^6^ and *I*_on_ > 100 µA.

Key Parameters of Pulse and FeFET Devices	Parameter Value/Range
Pulse amplitude	2.0~3.0 V, step size 0.2 V
Pulse width	1~11 μs, step size 2 μs
Activation field distribution *a*/*b*	2.3/0.4
Polarization *P*_r_	25 μC/cm^2^
Time constant *τ*_0_	1.9 × 10^−8^ s
Voltage acceleration factor *α*	3
Probability factor *β*	2
Thickness of the ferroelectric layer *T*_FE_	1.8 nm
Other MOS parameters	*N_A_* = 3 × 10^17^, *μ* = 50 cm^2^/(V·s), *V_D_* = 0.05 V

**Table 7:** Quasi-static interface-trap charge loading used for charge-matching sensitivity analysis.

*N*_it_ (cm^−2^)	∆*V*_FB_ (V)	*Q*_trap_ (µC/cm^2^)	|*Q*_trap_|/*P*_r_ (%)
0	0.000	0.000	0.00
1 × 10^12^	0.017	0.160	0.64
2 × 10^12^	0.033	0.320	1.28
5 × 10^12^	0.084	0.801	3.20

## Data Availability

The original contributions presented in this study are included in the article. Further inquiries can be directed to the corresponding author.
